# Exome sequencing of three cases of familial exceptional longevity

**DOI:** 10.1111/acel.12261

**Published:** 2014-08-12

**Authors:** Timothy P Cash, Guillermo Pita, Orlando Domínguez, Maria R Alonso, Leticia T Moreno, Consuelo Borrás, Leocadio Rodríguez-Mañas, Catalina Santiago, Nuria Garatachea, Alejandro Lucia, Juan A Avellana, Jose Viña, Anna González-Neira, Manuel Serrano

**Affiliations:** 1 Tumour Suppression Group; 2 Human Genotyping-CEGEN Unit; 3 Genomics Core Unit, Spanish National Cancer Research Centre (CNIO)Melchor Fernandez Almagro 3, 28029, Madrid, Spain; 4 Department of Physiology, School of Medicine, University of Valencia/INCLIVAAvda. Blasco Ibáñez 15, 46010, Valencia, Spain; 5 Department of Geriatrics, University Hospital of GetafeAutovía de Toledo km 11, Getafe, 28905, Madrid, Spain; 6 European UniversityC/Tajo s/n, Villaviciosa de Odón, 28670, Madrid, Spain; 7 Faculty of Health and Sport Science, University of ZaragozaRonda de Misericordia 5, 22001, Huesca, Spain; 8 Geriatric Unit, University Hospital RiberaCarretera Corbera km 1, Alzira, 46600, Valencia, Spain

**Keywords:** apolipoprotein B, centenarians, exome sequencing, longevity, rare variants

## Abstract

Exceptional longevity (EL) is a rare phenotype that can cluster in families, and co-segregation of genetic variation in these families may point to candidate genes that could contribute to extended lifespan. In this study, for the first time, we have sequenced a total of seven exomes from exceptionally long-lived siblings (probands ≥ 103 years and at least one sibling ≥ 97 years) that come from three separate families. We have focused on rare functional variants (RFVs) which have ≤ 1% minor allele frequency according to databases and that are likely to alter gene product function. Based on this, we have identified one candidate longevity gene carrying RFVs in all three families, *APOB*. Interestingly, APOB is a component of lipoprotein particles together with APOE, and variants in the genes encoding these two proteins have been previously associated with human longevity. Analysis of nonfamilial EL cases showed a trend, without reaching statistical significance, toward enrichment of *APOB* RFVs. We have also identified candidate longevity genes shared between two families (5–13) or within individual families (66–156 genes). Some of these genes have been previously linked to longevity in model organisms, such as *PPARGC1A*,*NRG1*,*RAD52, RAD51, NCOR1*, and *ADCY5* genes. This work provides an initial catalog of genes that could contribute to exceptional familial longevity.

While there is no doubt that environment impacts longevity, genetics also plays an important role. Single gene manipulations in organisms from yeast to mice can result in lifespan extension (Kuningas *et al*., 2008). In addition, twin studies have shown that genetics explains 25% of variation in survival into the ninth decade of life, and this influence becomes more significant in cases of extreme longevity (EL, ≥ 100 years of age), which is a rare phenotype, occurring in only 1/5000 individuals (Herskind *et al*., 1996; Perls *et al*., 2007; Westendorp *et al*., 2009; Sebastiani & Perls, 2012). It has also been demonstrated that EL is heritable with the observation that siblings of centenarians having an eightfold to 17-fold increased chance of reaching 100 years of age for females and males, respectively (Perls *et al*., 2002). Common coding variants in *APOE* and common noncoding variants in *FOXO3A* have been associated with EL and replicated across multiple populations (Deelen *et al*., 2011; Brooks-Wilson, 2013; Garatachea *et al*., 2014). Further, a previous study identified a complex signature of 281 common genetic variants that can be used to predict EL (Sebastiani *et al*., 2012). However, much less is known about the contribution of rare variants to EL.

To identify rare variants associated with EL, we initiated a pilot study in which we exome-sequenced three different families where EL clustered. An analogous approach is widely being employed to identify disease-causing genes associated with rare familial syndromes (Wang *et al*., 2013). For our study, we selected probands (ages ≥ 103) from three separate families in Spain, referred to as Families A, B, and C, which included siblings approaching or over 100 years of age. The proband from Family A (lived to age 104, now deceased) has two living siblings with current ages 97 and 94 years, that from Family B (currently age 103 years) has one living sibling of age 97 years, and the proband from Family C (lived to age 103 years, now deceased) has one living sibling of age 100 years and one sibling who had died at age 101 years (Fig.1A). We isolated peripheral blood DNA from the probands and their surviving siblings and performed exome capture and second-generation sequencing.

**Figure 1:**
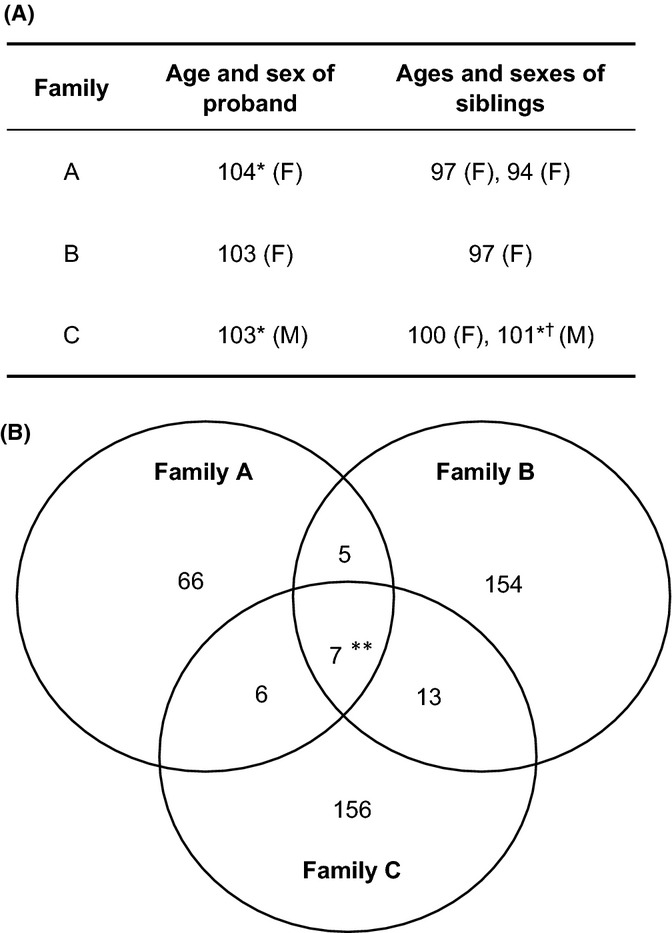
Rare functional variants (RFVs) in cases of familial extreme longevity (EL). (A) Table showing the ages (in years) and genders (F = female, M = male) of individuals in the 3 families, designated Families A, B, and C, where EL clusters (*indicates those individuals who are deceased, and ^†^ indicates an individual who was not used in this study). (B) Venn diagram depicting the number of genes containing RFVs shared by all the members of a family. (** for variants shared by all families indicates the presence of a high number of likely false positives in this group, see main text).

To identify potential rare candidate variants that might contribute to familial EL, we focused on variants that were of very low frequency and likely to have functional consequences. First, we only considered variants that had ≤ 1% or unknown minor allele frequency (MAF) according to publicly available databases and were not present in nine fully sequenced HAPMAP controls aligned in house. Second, we only considered gene variations that altered the open reading frame of the encoded protein (nonsynonymous amino acid changes, frameshifts, truncations, nonframeshifting indels, splice site alterations and stop codon losses). We refer to these variants of low (≤ 1% MAF) or unknown frequency and having potentially functional impact as ‘rare functional variants’ (RFVs).

Following the above filtering criteria, we obtained 1774 RFVs across all seven sequenced exomes, with an average of 432 RFVs per individual (Table S1). We did not find any genes carrying RFVs in homozygosity that were shared across all families. However, we found seven genes carrying a total of 14 RFVs in heterozygosity that were shared across all three families (Fig.1B; Fig. S1). Out of the seven genes, we considered six of them to be of low interest either because variants in these genes are known to be common false positives in exome sequencing studies (*PCMTD1*,*PRUNE2*,*MUC16*,*MUC6* and *CDC27*) (Fuentes Fajardo *et al*., 2012) or because their described biological function is unlikely to contribute to human longevity (e.g., *KLK4* which plays a role in tooth enamel formation) (Hu & Simmer, 2007). The exception was the apolipoprotein B (*APOB*) gene, which encodes a component of circulating lipoproteins.

Interestingly, reciprocal associations between hypolipobetaproteinemia, a condition caused by *APOB* mutations, and increased longevity have been described (Glueck *et al*., 1976; Arai *et al*., 1997). Also, common variants in *APOE*, which encodes another component of circulating lipoproteins, are irrefutably associated with longevity (Deelen *et al*., 2011). All three members of Family A harbored the *APOB* variant rs1801703, and all members of Families B and C carried 2 *APOB* variants rs12713450 and rs12720854, which appeared to be in linkage disequilibrium in the Spanish control population (*r* = 0.759, D’ = 1.000), and these three RFVs were confirmed by Sanger sequencing. To assess whether these *APOB* variants might be more broadly associated with EL, we genotyped 203 nonfamilial cases of EL from Spain together with Spanish control individuals (*n *= 764). While the MAFs for all 3 *APOB* variants were higher in the nonfamilial EL cohort than in the Spanish control group, the differences did not reach statistical significance (Fig.2A). We further hypothesized that perhaps nonfamilial cases of EL might harbor variants that differed from those found in familial EL or might carry a higher RFV burden than the control population. To assess this possibility, we Sanger-sequenced *APOB* exons 26 and 29, which comprise 69% (9.5 kB) of the gene's coding region, in 47 cases of nonfamilial EL and 47 Spanish controls. We focused on these exons because they encode the LDL receptor-binding domain and the auto-regulatory C-terminal tail. Also, more than two-thirds of mutations associated with hypobetalipoproteinemia, a proposed ‘longevity syndrome’, as well as the variants identified in our familial cases of EL, lie within these exons (Whitfield *et al*., 2004). Altogether, 8 of 47 EL individuals carried a total of seven different *APOB* RFVs, while 7 of 47 controls harbored a total of 6 different *APOB* RFVs, which did not reach statistical significance by SKAT analysis (*P *=* *0.16, Fig.2B). While the findings in this pilot study do not demonstrate an association between the number of RFVs in *APOB* exons 26 and 29 in EL individuals vs. controls, sequencing of additional exons and analysis of larger EL populations will be required to unequivocally determine this.

**Figure 2:**
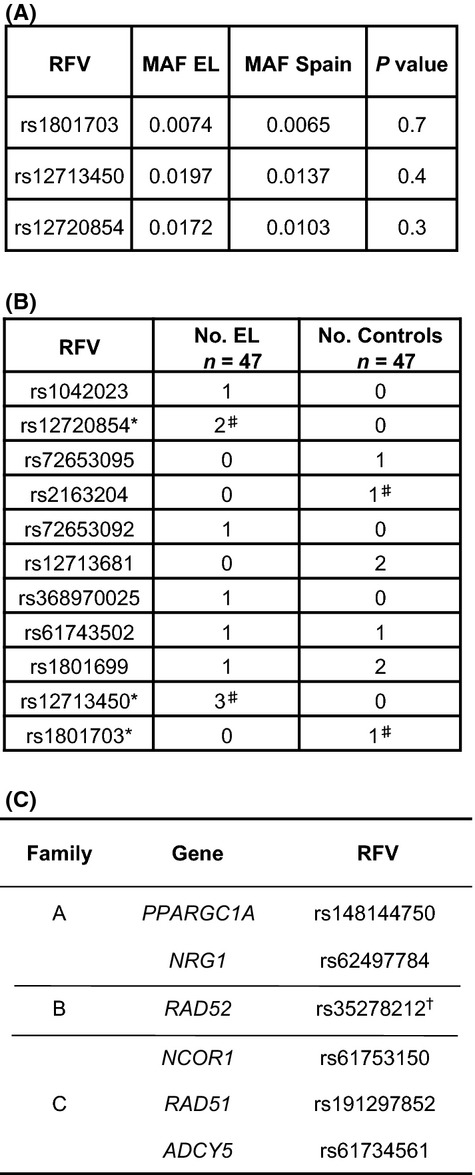
(A) Table showing the minor allele frequencies (MAFs) for the indicated rare functional variants (RFVs) in nonfamilial EL (*n *= 203) and in the Spanish control group (*n *= 764). Exact numbers of informative genotypes for each variant can be found in Data S1. (B) Table of RFVs in *APOB* exons 26 and 29, in 47 cases of nonfamilial EL individuals, or 47 controls. * indicates variants found in familial cases of EL (see Fig.2A). ^#^ indicates variants that were found in the same individual: rs12720854 and rs12713450 were found in the same 2 EL individuals; and rs2163204 and rs1801703 were both found in the same control individual. (C) Table of genes and associated RFVs present within each family that are found in the HAGR database for genes associated with aging and longevity in human. ^†^ indicates that the *RAD52* variant occurs within a *RAD52* splice variant that is annotated as ‘protein coding’ in ENSEMBL, but is not included in the Consensus CDS protein set.

In addition to RFVs in *APOB*, which were found in individuals across all three families with EL, we also found RFVs shared among siblings within individual families. We only found one RFV that was homozygous among all siblings of a family (Family C). This was the case of rs71549220 in *HLA-DRB5* which encodes an MHCII subunit and which has uncertain relevance to longevity. We found a total of 400 genes carrying RFVs in heterozygosity shared by all the siblings of a given family (including a few genes that were shared among two families) (Fig.1B and Table S2 and S3). To identify which of these genes might be promising candidates for further investigation, we utilized the Human Ageing Genomic Resources (HAGR) search engine for aging-related human genes (de Magalhaes *et al*., 2009). Two genes from Family A (*PPARGC1A* and *NRG1*), 1 gene from Family B (*RAD52*), and 3 genes from Family C (*RAD51, NCOR1*, and *ADCY5*) were identified in the HAGR database as having a possible association with human aging and longevity (Fig.2C). Several more genes containing RFVs were identified in the HAGR database for genes associated with aging and longevity in model organisms (Fig. S2).

In this pilot study, for the first time, we have used exome sequencing to identify RFVs present in families where EL clusters. While we currently cannot make any strong claims about genotype–phenotype correlations regarding cases of familial EL, this work is a starting point and a proof-of-principle that such an approach can be employed to narrow down lists of candidate genes contributing to human EL, analogous to that used to pinpoint disease-causing mutations in rare Mendelian disorders. Additionally in this study, we have uncovered rare functional variants in the *APOB* gene, which are present in multiple cases of familial EL, and we have generated a catalog of hundreds of RFVs that may contribute to extended lifespan in individual families. Validation of the allele frequency of these variants in larger cohorts of nonfamilial cases of EL, as well as functional validation of biological effects, is currently underway to further understand their relative contributions to human EL.

## Supporting Information

**Fig. S1** Genes containing functional rare variants that are common across all 7 individuals from the 3 families. **Fig. S2** Genes containing rare functional variants present in the GenAge Database of Ageing-Related Genes for model organisms by family.

**Table S1** Variants of unknown frequency and of frequency ≤1% in HAPMAP CEU or 1000G CEU when HAPMAP frequency was not available, identified in at least 1 of the 7 sequenced individuals in Families A, B and C. **Table S2** Genes that contain rare functional variants shared among all the members of each family. **Table S3** Genes that contain rare functional variants shared between two of the three families.
