# Supplementary material for: Exome sequencing of three cases of familial exceptional longevity
Source: Aging Cell. 2014 Aug 12;13(6):1087–90. doi: 10.1111/acel.12261 (PMC4326919; doi:10.1111/acel.12261)
Supplement: Supplementary file 1 — Fig. S1 Genes containing functional rare variants that are common across all 7 individuals from the 3 families. Fig. S2 Genes containing rare functional variants present in the GenAge Database of Ageing-Related Genes for model organisms by family. [file acel0013-1087-sd1.pdf]

Supplementary Figure 1

Gene	DBsnp Id/chr:position	Ref/Var Allele	Family where present
APOB	rs12713450	G/A	B and C
APOB	rs1801703	C/T	A
APOB	rs12720854	T/C	B and C
PCMTD1	rs79195845	C/T	A, B and C
PCMTD1	rs75865149	A/C	A, B and C
PRUNE2	rs11267615	GT/G	A, B and C
MUC6	rs75538227	G/A	A
MUC6	rs78003962	G/C	B and C
MUC6	chr11:1018419	G/A	B and C
MUC6	chr11:1017768	G/A	B
MUC6	rs115755410	G/A	B and C
CDC27	chr17:45234303	G/C	A, B and C
MUC16	chr19:9005689	G/A	A, B and C
KLK4	chr19:51412012	T/TGGGGG	A, B and C

Supplementary Figure 2

Family A	Family B	Family C
ATG12	*ABCC12*	*ACVR1*
HSPG2	*CNGA4*	*AKAP1*
KIF17	*EXOSC3*	*ATG2A*
NUP85	*GDI2*	*CYP2D6*
RNF157	*NOB1*	*CYP3A43*
ZNF496	*PABPC1*	*FLNB*
	RAB5A	*IFT172*
	RRAS	*PABPC1*
	SLC25A5	*SLC25A5*
	ZNF444	
