# Supplementary material for: Exome sequencing of three cases of familial exceptional longevity
Source: Aging Cell. 2014 Aug 12;13(6):1087–90. doi: 10.1111/acel.12261 (PMC4326919; doi:10.1111/acel.12261)
Supplement: Data S1 — Materials and Methods. [file acel0013-1087-sd3.docx]

**Supplementary Figure Legends**

**Supplementary Figure 1:** Genes containing functional rare variants that are common across all 7 individuals from the 3 families. The corresponding variant positions, base pair changes and families where present are indicated. Most of these variants represent likely false positives with the notable exception of *APOB*, which was Sanger sequence confirmed.

**Supplementary Figure 2**: Genes containing rare functional variants present in the GenAge Database of Ageing-Related Genes for model organisms by family.

**Supplementary Table Legends**

**Supplementary Table 1:** Variants of unknown frequency and of frequency ≤1% in HAPMAP CEU or 1000G CEU when HAPMAP frequency was not available, identified in at least 1 of the 7 sequenced individuals in Families A, B and C. “0/0” denotes absence of a rare variant, “0/1” denotes presence of a variant in heterozygous state and “1/1” denotes presence of a variant in the homozygous state. “.” denotes base calls that were undetermined in the exome sequencing. dbSNP identifier, chromosomal position, base quality score, gene name, effect on amino acid sequence, etc. is also noted in the table. Amino acid changes are listed for the open reading frame of one transcript at each base position. Other amino acid changes for other transcripts and reading frames may be annotated for some variants in ENSEMBL.

**Supplementary Table 2:** Genes that contain rare functional variants shared among all the members of each family.

**Supplementary Table 3**: Genes that contain rare functional variants shared between two of the three families.

**Materials and Methods**

**Cases and controls**

All exome-sequenced individuals resided in Comarca de la Ribera (Valencia, Spain) and all blood samples from these individuals were obtained and processed in the University Hospital Ribera (Alzira, Spain). The study was approved by the Ethical and Clinical Trials Committee at the University Hospital Ribera. For assessing the frequency of variants in sporadic cases of EL, 24 EL samples from Alzira were obtained from University Hospital Ribera and 21 more from the Spanish National DNA Bank (Salamanca, Spain, [http://www.bancoadn.org](https://mail.cnio.es/owa/redir.aspx?C=G4h9tAi6pEyXbK0h-pGT7t1GPtWoCdEIKfyKSwUHSpfbovM5g7Wu_Rxw-Tx4joBzNCvZUSFIMdE.&URL=http%3a%2f%2fwww.bancoadn.org)) which represented individuals from all over Spain. Additional DNA samples from 161 EL individuals, residing mostly in the central area of Spain (Castilla-León, Castilla-La Mancha, Community of Madrid), were obtained from the European University, Madrid, Spain. The total cohort of sporadic cases was composed of 166 females and 40 males. The table below shows the distribution of ages for all individuals.

| **Age** | **Number of Individuals** |
| --- | --- |
| 98 | 1 |
| 99 | 18 |
| 100 | 43 |
| 101 | 50 |
| 102 | 38 |
| 103 | 23 |
| 104 | 8 |
| 105 | 10 |
| 106 | 6 |
| 107 | 5 |
| 108 | 3 |
| 111 | 1 |

All of these individuals were given written informed consent and approval was obtained from the IRBs of the aforementioned institutions. The Spanish controls were composed of 556 women and 351 men.

**Exome capture and sequencing**

DNA samples were enriched for exomic regions according to Agilent's SureSelect Human All Exon 50 Mb Kit protocol (Agilent Technologies). Resulting DNA libraries were sequenced using 78-bp paired-end technology on an Illumina Genome Analyzer II following the manufacturer's protocol using 1 sequencing lane per sample. Real-time analysis and base calling was performed using Illumina's Real Time Analysis software version 1.6 using standard parameters**.**

**Sequencing data analysis**

Obtained reads were mapped against the human reference genome (UCSC

hg19/GRCh37) and read pairing was performed with the Burrows-Wheeler Aligner (BWA) (Li & Durbin 2009). The SAM files were converted to BAM files and duplicate molecules were marked with picard-tools v1.5.1 (<http://picard.sourceforge.net>). Base quality score recalibration, indel realignment, duplicate removal, and SNP and INDEL discovery were performed using GATK v.1.3 (McKenna *et al.* 2010), and genotyping across all samples simultaneously using standard hard filtering parameters or variant quality score recalibration (DePristo *et al.* 2011). Variants having a base quality score less than 30 were removed. All variants were annotated using snpEff v2.0.2 (Cingolani *et al.* 2012) and filtered using a developed Perl script. Exome data from nine HAPMAP samples from the CEU population (NA06984, NA06986, NA06989, NA06994, NA07037, NA07048, NA07051, NA07056, NA07347) was downloaded from the 1000G project (<ftp://ftp.1000genomes.ebi.ac.uk/vol1/ftp/data/>) and was aligned and filtered in-house. Variants considered rare (listed in Supplementary Table 1) were those with ≤1% frequency in the HAPMAP CEU population or ≤1% frequency in the 1000G CEU population when HAPMAP frequency was not available. All variants within unknown frequency in both HAPMAP CEU and 1000G CEU were considered rare and included in Table 1. The number of RFVs for each of the 7 individuals is listed below before and after each filter. Total number of RFVs for each individual after all filters fall within an expected range according to previous reports (Abecasis *et al.* 2012).

|  | ***Fam A*** | | | ***Fam B*** | | ***Fam C*** | |
| --- | --- | --- | --- | --- | --- | --- | --- |
|  | ***Proband*** | ***Sib (97)*** | ***Sib (94)*** | ***Proband*** | ***Sib*** | ***Proband*** | ***Sib*** |
| **Total known freq** | 22006 | 27726 | 27755 | 27140 | 27452 | 26448 | 26839 |
| **MAF ≤.01** | 268 | 364 | 345 | 360 | 350 | 335 | 333 |
| **High or med effect** | 133 | 172 | 176 | 160 | 169 | 172 | 151 |
| **Protein coding only** | 102 | 133 | 126 | 121 | 124 | 123 | 113 |
| **Absent in 9 HAPMAP** | 76 | 113 | 106 | 94 | 92 | 95 | 86 |
|  |  |  |  |  |  |  |  |
| **Unknown freq** | 7657 | 10210 | 10401 | 10015 | 9834 | 9795 | 9973 |
| **High or med effect** | 2615 | 3524 | 3548 | 3367 | 3256 | 3247 | 3314 |
| **Protein coding only** | 1658 | 2121 | 2161 | 1973 | 1825 | 1916 | 1985 |
| **Absent in 9 HAPMAP** | 304 | 361 | 368 | 330 | 317 | 335 | 349 |
| **Total RFVs** | **380** | **474** | **474** | **424** | **409** | **430** | **435** |

**KASPar assays**

Genotyping for APOB variants was carried out using KASPar assays according to the manufacturer's protocol (KBioscience, Beverly, MA, USA). For EL cases, 206 individuals of ≥100 years of age were analyzed. For control populations, Spanish individuals were genotyped for each single nucleotide variant in *APOB*. Below is the number of successful, informative genotypes obtained for each assay for each of the populations.

| **SNV** | **EL** | **Spain** |
| --- | --- | --- |
| rs1801703 | 203 | 768 |
| rs12713450 | 203 | 764 |
| rs12720854 | 203 | 770 |

**Statistics**

Statistical significance and *p* values for EL MAFs vs. control populations in Spain were determined using a Fisher’s two-sided exact test. SKAT analysis was done using the standard SNP-set (sequence) kernel association test package with default parameters ) kernel="linear.weighted", method="davies", weights.beta= c(1,25)), considering a binary trait and no covariate were included.

**Sanger sequencing**

APOB exons were PCR amplified as previously described (Tarugi *et al.* 2001) and sequenced on an ABI 3730xl sequencer. We acquired informative sequence for about 96% of the total exonic sequence in exons 26 and 29. All sequences were manually inspected to identify variants.

**Use of Human Ageing Genomic Resources (HAGR)**

Genes associated with rare functional variants found within each family (those found in Supplementary Tables 3 and 4) were queried in the “human gene” and “model organism” search tool of the HAGR database.

**References:**

Abecasis GR, Auton A, Brooks LD, DePristo MA, Durbin RM, Handsaker RE, Kang HM, Marth GT , McVean GA (2012). An integrated map of genetic variation from 1,092 human genomes. *Nature*. **491**, 56-65.

Cingolani P, Platts A, Wang le L, Coon M, Nguyen T, Wang L, Land SJ, Lu X , Ruden DM (2012). A program for annotating and predicting the effects of single nucleotide polymorphisms, SnpEff: SNPs in the genome of Drosophila melanogaster strain w1118; iso-2; iso-3. *Fly (Austin)*. **6**, 80-92.

DePristo MA, Banks E, Poplin R, Garimella KV, Maguire JR, Hartl C, Philippakis AA, del Angel G, Rivas MA, Hanna M, McKenna A, Fennell TJ, Kernytsky AM, Sivachenko AY, Cibulskis K, Gabriel SB, Altshuler D , Daly MJ (2011). A framework for variation discovery and genotyping using next-generation DNA sequencing data. *Nat Genet*. **43**, 491-498.

Li H , Durbin R (2009). Fast and accurate short read alignment with Burrows-Wheeler transform. *Bioinformatics*. **25**, 1754-1760.

McKenna A, Hanna M, Banks E, Sivachenko A, Cibulskis K, Kernytsky A, Garimella K, Altshuler D, Gabriel S, Daly M , DePristo MA (2010). The Genome Analysis Toolkit: a MapReduce framework for analyzing next-generation DNA sequencing data. *Genome Res*. **20**, 1297-1303.

Tarugi P, Lonardo A, Gabelli C, Sala F, Ballarini G, Cortella I, Previato L, Bertolini S, Cordera R , Calandra S (2001). Phenotypic expression of familial hypobetalipoproteinemia in three kindreds with mutations of apolipoprotein B gene. *J Lipid Res*. **42**, 1552-1561.
